# Efficacy and safety of teneligliptin added to metformin in Chinese patients with type 2 diabetes mellitus inadequately controlled with metformin: A phase 3, randomized, double‐blind, placebo‐controlled study

**DOI:** 10.1002/edm2.222

**Published:** 2021-01-20

**Authors:** Linong Ji, Ling Li, Jianhua Ma, Xuefeng Li, Dongmei Li, Bangzhu Meng, Weiping Lu, Jiao Sun, Yanmei Liu, Gen Takayanagi, Yi Wang

**Affiliations:** ^1^ Peking University People's Hospital Beijing China; ^2^ Shengjing Hospital of China Medical University Liaoning China; ^3^ Nanjing First Hospital Nanjing Jiangsu China; ^4^ Shiyan Taihe Hospital Hubei China; ^5^ Inner Mongolia People’s Hospital Inner Mongolia China; ^6^ The Affiliated Hospital of Inner Mongolia University for Nationalities Inner Mongolia China; ^7^ Huai'an First People’s Hospital Nanjing Medical University Nanjing Jiangsu China; ^8^ Huadong Hospital Affiliated to Fudan University Shanghai China; ^9^ Yancheng City No.1 People's Hospital Jiangsu China; ^10^ Mitsubishi Tanabe Pharma Development America, Inc. Jersey City NJ USA; ^11^ Mitsubishi Tanabe Pharma Development (Beijing) Co., Ltd. Beijing China

**Keywords:** diabetes mellitus, dipeptidyl peptidase‐IV inhibitors, metformin, type 2

## Abstract

**Introduction:**

We evaluated the efficacy and safety of teneligliptin compared with placebo when added to metformin therapy in Chinese patients with type 2 diabetes inadequately controlled with metformin monotherapy.

**Methods:**

This multicentre, randomized, double‐blind, placebo‐controlled, parallel‐group study enrolled type 2 diabetes patients with glycosylated haemoglobin (HbA1c) 7.0%−<10.0% and fasting plasma glucose (FPG) <270 mg/dl, receiving a stable metformin dose ≥1000 mg/day. Teneligliptin 20 mg or placebo was administered orally once daily (qd) before breakfast for 24 weeks. The primary efficacy end‐point was change in HbA1c from baseline to Week 24. Safety end‐points included the incidence of adverse events (AEs).

**Results:**

The least square mean (LSM) change from baseline (standard error [SE]) was −0.72 (0.07) (95% confidence intervals [CI], −0.87, −0.58) for teneligliptin and −0.01 (0.07) (95% CI, −0.16, 0.13) for placebo. The differences (LSM ± SE) between the placebo and teneligliptin groups in HbA1c and FPG were −0.71% ± 0.11% (*p* < .0001) and −16.5 ± 4.7 mg/dl (*p* = .0005), respectively. Teneligliptin yielded significant changes in HbA1c (−0.81%; *p* < .0001) and FPG (−22.2 mg/dl; *p* < .0001) at Week 12. At Week 24, more patients achieved HbA1c <7.0% with teneligliptin (41.7%) compared with placebo (16.1%; *p* < .0001). Treatment‐emergent AE incidence was similar with teneligliptin (58.9%) and placebo (68.3%); upper respiratory tract infection, hyperuricaemia and hyperlipidaemia were the most common AEs.

**Conclusions:**

Teneligliptin 20 mg qd for 24 weeks added to ongoing metformin treatment significantly decreased HbA1c and FPG levels compared with placebo in Chinese type 2 diabetes patients. The combination was safe and tolerable.

## Graphical abstract

In this multicenter, randomized, double‐blind, placebo‐controlled, parallel‐group study, we evaluated the efficacy and safety of teneligliptin compared with placebo when added to metformin therapy in Chinese patients with type 2 diabetes inadequately controlled with metformin monotherapy. Eligible type 2 diabetes patients receiving a stable metformin dose ≥1000 mg/day received teneligliptin 20 mg or placebo orally once daily (qd) before breakfast for 24 weeks to assess the primary efficacy end‐point (change in HbA1c from baseline to Week 24) and safety (including the incidence of adverse events). The least square mean (LSM) change from baseline (standard error [SE]) was −0.72 (0.07) for teneligliptin and −0.01 (0.07) for placebo, and the differences (LSM ± SE) between the placebo and teneligliptin groups in HbA1c and FPG were −0.71% ± 0.11% and −16.5 ± 4.7 mg/dl, with similar treatment‐emergent AE incidences in both groups, indicating that teneligliptin 20 mg qd for 24 weeks added to ongoing metformin treatment was safe and tolerable, and significantly decreased HbA1c and FPG levels compared with placebo in Chinese type 2 diabetes patients.

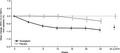

## 1 INTRODUCTION

China is the most populous country in the world and has the largest population of diabetes patients.^1^ It is estimated that over 100 million Chinese individuals have diabetes.^1^ The sharp increase in diabetes prevalence in China in the past 30 years^1^ has raised many concerns and emphasizes the need for more stringent prevention and treatment strategies.

Aside from traditional lifestyle changes and initial first‐line treatment with metformin for patients inadequately controlled with lifestyle changes alone, current management guidelines recommend intensification of treatment with other antihyperglycaemic agents.^2^, ^3^ With prolonged use and disease progression, metformin monotherapy may be less effective for disease control.^4^

The burden of type 2 diabetes is growing, with long‐term microvascular (ie, nephropathy, neuropathy and retinopathy), macrovascular (eg, atherosclerosis and peripheral vascular diseases) and other complications.^5^, ^6^ Additionally, current standard treatments have several limitations, such as poor medication adherence,^7^ hypoglycaemia, weight gain and treatment refractoriness. This has led to the development of new classes of antihyperglycaemic agents, such as dipeptidyl peptidase (DPP)‐4 inhibitors.

DPP‐4 inhibitors have shown efficacy in improving glucose control; they lower glycosylated haemoglobin (HbA1c) levels by reducing both fasting and postprandial glucose levels, without causing weight gain, hypoglycaemia, or other relevant adverse events (AEs).^8^, ^9^ DPP‐4 inhibitors can be used as monotherapy and in combination with other agents with complementary mechanisms of action, such as metformin. Such combinations result in increasing concentrations of active glucagon‐like peptide 1 (GLP‐1),^10^, ^11^ which has an insulinotropic effect and glucagonostatic actions that can augment postprandial insulin secretion, resulting in a glucose‐lowering effect.^12^, ^13^, ^14^

Teneligliptin is a potent third‐generation DPP‐4 inhibitor with long action duration that results in stable glucose levels during the day^15^, ^16^ and inhibitory effects lasting 24 h.^17^ Teneligliptin requires no dose adjustment because of hepatic and renal excretion,^16^ even in patients with severe renal impairment or end‐stage renal disease.^18^ Furthermore, it has pleiotropic effects, including improvements in lipid profile, left ventricular function, adiponectin levels and a natriuretic effect.^17^

Previous studies of DPP‐4 inhibitors in combination with metformin ^19^, ^20^, ^21^ as well as studies of teneligliptin added to metformin therapy^22^, ^23^ conducted elsewhere showed that the combination was generally tolerable and resulted in improved glucose control, without increased hypoglycaemic risk. However, no clinical trials of teneligliptin added to metformin therapy in type 2 diabetes patients inadequately controlled with metformin monotherapy have been conducted in China. This study evaluated the efficacy and safety of teneligliptin compared with placebo when added to metformin therapy in Chinese patients with type 2 diabetes inadequately controlled with metformin monotherapy, diet and exercise.

## 2 MATERIALS AND METHODS

### 2.1 Study design, setting, randomization and blinding

This was a multicentre, randomized, double‐blind, placebo‐controlled, parallel‐group study (NCT02924064) conducted in 51 sites in China. The study had a 30‐week duration, including 2‐week screening, 2‐week placebo run‐in, 24‐week treatment and 2‐week follow‐up periods (Figure S1). Patients had six additional clinic visits (at weeks 4, 8, 12, 16, 20 and 24) and a follow‐up telephone visit at Week 26.

Patients with 75% or higher treatment compliance during the placebo run‐in period were randomly assigned to teneligliptin 20 mg once daily or placebo in a 1:1 ratio by a computer‐generated randomization code. The Interactive Web Randomization System was used for static block randomization. Patients, investigators, laboratory personnel and sponsors were blinded to treatment.

Patients were free to discontinue their participation in the study by withdrawing consent or could be withdrawn from the study at any time if they presented with lack of glycaemic control during the double‐blind treatment period, onset of health‐endangering AEs, deterioration of their medical condition and requiring therapy/treatment, or by investigator's decision.

### 2.2 Participants

Patients with a documented diagnosis of type 2 diabetes; age ≥18 years; with an HbA1c value ranging from ≥7.0% to <10.0% and fasting plasma glucose (FPG) <270 mg/dl (15 mmol/L) at the screening visit (Day −28) and on Day −14; and undergoing a stable regimen (ie, used during the 8 weeks prior to study start) of metformin monotherapy ≥1000 mg/day plus diet and exercise therapy, which remained unchanged for at least eight consecutive weeks at the screening visit (Day −28), were enrolled in this study.

Patients were excluded from the study if they had a history of type 1 diabetes or a secondary form of diabetes; previous insulin treatment within 1 year prior to the screening visit; treatment with any prohibited concomitant medication within 8 weeks prior to the screening; and comorbidities. Further exclusion criteria are provided in Appendix S1.

### 2.3 Interventions

The treatment intervention in this study consisted of the administration of teneligliptin (Mitsubishi Tanabe Pharma Corporation) at a dose of 20 mg or placebo (formulated and packaged identically to the active drug), both administered orally once daily before breakfast for 24 weeks in patients already receiving monotherapy with a stable dose of metformin ≥1000 mg/day. The prohibited concomitant medications were insulin, sulfonylureas, alpha‐glucosidase inhibitors, thiazolidinediones, glinides, DPP‐4 inhibitors, GLP‐1 receptor agonists, herbal medicines that lower blood glucose levels, new drugs intended for diabetes, or fixed‐dose combination tablets including the above‐mentioned active ingredients, and adrenocorticosteroids (excluding for external use). Medications not mentioned here could be used concomitantly for the treatment of complications and AEs. In principle, medications already in use at the screening visit were used until 2 weeks after the final dose of the study drug without any change in prescription. No dose adjustments were planned.

### 2.4 Outcomes

The primary efficacy end‐point was change in HbA1c from baseline to Week 24.

The secondary efficacy end‐point was change in FPG from baseline to Week 24.

Other end‐points were as follows: proportion of patients who achieved HbA1c <7.0% at Week 24; change in fasting insulin, C‐peptide and glucagon from baseline to Week 24; change in homeostatic model assessment‐insulin resistance and homeostatic model assessment‐beta‐cell function (HOMA‐IR and HOMA‐β) from baseline to Week 24; change in body weight from baseline to Week 24; and change in HbA1c and FPG from baseline to Week 12.

The safety end‐points were AEs, classified using the Medical Dictionary for Regulatory Activities version 21.0; adverse drug reactions (ADRs); treatment‐emergent AEs (TEAEs); hypoglycaemic episodes; cardiovascular events (adjudicated by an independent event adjudication committee); vital sign measurements (blood pressure, pulse rate and body temperature); laboratory measurements; and 12‐lead electrocardiogram (ECG).

### 2.5 Measures

Data on baseline demographic and disease characteristics of patients were collected. Blood samples to measure HbA1c, FPG, insulin, C‐peptide and glucagon were collected in a fasted state at study sites and were measured at a central laboratory. Documented symptomatic hypoglycaemia is defined in Appendix S1. Cardiovascular events (ie, death, myocardial infarction, hospitalization for unstable angina or heart failure, stroke or transient ischaemic attack, or urgent revascularization procedures) were evaluated by an event adjudication committee.

### 2.6 Statistical methods

#### 2.6.1 Sample size

The planned sample size was 240 patients, with 120 patients to be randomly assigned to each treatment group. Further details are provided in Appendix S1.

#### 2.6.2 Statistical analysis

The analysis sets are defined in Appendix S1. Efficacy and safety analyses were performed using the full analysis set (FAS) and safety analysis set, respectively.

All statistical tests were 2‐sided with a significance level of 5%; 95% confidence intervals (CI) were calculated for the treatment effects and differences between groups. Descriptive statistics (number of non‐missing values [*n*], mean, standard deviation [SD], median, minimum and maximum) were used for continuous variables and frequency counts and percentages for discrete variables.

Analysis of covariance (ANCOVA) was used for the analysis of the primary, secondary and other efficacy end‐points, with treatment as a fixed effect and baseline as a covariate; that is, ANCOVA was used to adjust for differences in the baseline HbA1c value between treatment groups during the analysis of the primary efficacy end‐point (ie, change in HbA1c from baseline to Week 24). Prior to the analysis, missing values at Week 12 or 24 were imputed using the last observation carried forward (LOCF) method. Additionally, the mixed‐effects model for repeated measures (MMRM) was used to assess the robustness of the results of the primary analysis, with treatment, visit, and interaction of treatment and visit as fixed effects, and baseline as a covariate. For the proportion of patients who achieved HbA1c <7.0% at Week 24 (LOCF), a logistic regression analysis was performed with treatment as a fixed effect and baseline HbA1c value as a covariate. Subgroup analyses were conducted for change in HbA1c from baseline to Week 24 by baseline characteristics. Multiplicity due to multiple testing was not adjusted within or between the primary, secondary and other efficacy end‐points. SAS Version 9.2 or higher (SAS Institute) was used for the analyses.

### 2.7 Ethical considerations

The protocol and other related documents were approved by an Independent Ethics Committee. The study was conducted according to the 2013 (Fortaleza) revision of the Declaration of Helsinki, Good Clinical Practice as required by the International Council on Harmonisation guidelines, and all applicable regional and local legislation.

## 3 RESULTS

### 3.1 Participants

**FIGURE 1:**
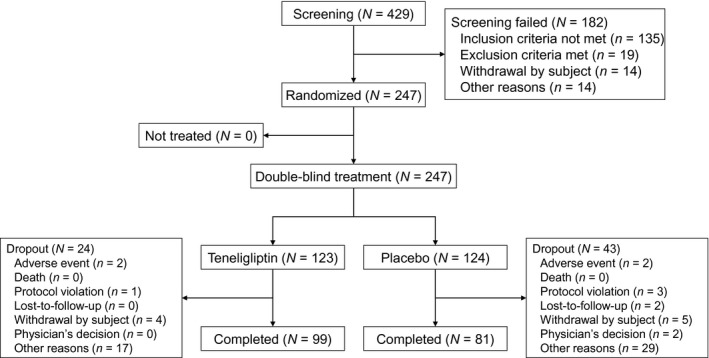
Patient disposition

Of the total of 429 patients who provided informed consent, 247 patients were randomly assigned to treatment (123 patients to the teneligliptin group and 124 to the placebo group); 182 patients were not eligible for randomization, the majority of whom did not meet inclusion criteria (*n* = 135). The remainder either met exclusion criteria (*n* = 19), withdrew from participation (*n* = 14), or had other reasons (*n* = 14) (Figure 1). Of the 247 randomly assigned patients, 180 patients (72.9%) completed the study, including 99/123 (80.5%) patients in the teneligliptin group and 81/124 (65.3%) in the placebo group. In the teneligliptin group, the main reasons for premature study discontinuation were other reasons (*n* = 17, of which 15 were due to lack of glycaemic control), patient withdrawal (*n* = 4) and AEs (*n* = 2). In the placebo group, the main reasons were other reasons (*n* = 29, of which 28 were due to lack of glycaemic control), patient withdrawal (*n* = 5), protocol violation (*n* = 3), and lost to follow‐up, AEs, and physician's decision (*n* = 2 each).

### 3.2 Baseline data

Baseline characteristics of both groups were generally comparable (Table 1). In the teneligliptin and placebo groups (FAS), respectively, 66.4% and 54.0% of patients were male, with a mean ± SD age of 56.0 ± 9.8 and 54.7 ± 10.1 years, and a mean ± SD body mass index (BMI) of 26.00 ± 3.15 and 26.19 ± 3.22 kg/m^2^. The mean ± SD duration of diabetes was 5.05 ± 3.90 and 5.41 ± 4.22 years, the mean ± SD of HbA1c at baseline was 7.90 ± 0.68% and 7.87 ± 0.72%, and the mean ± SD of metformin total daily dose was 1368.9 ± 341.4 and 1392.9 ± 353.1 mg, respectively. The treatment compliance was greater than 75% in 98.4% of patients in the teneligliptin group and in 99.2% of patients in the placebo group.

**TABLE 1:** Baseline characteristics of patients.

	Teneligliptin (*N* = 122)	Placebo (*N* = 124)
Age (years), mean (SD)	56.0 (9.8)	54.7 (10.1)
Median (range)	57.0 (28, 75)	55.0 (28, 76)
Sex, *n* (%)		
Male	81 (66.4)	67 (54.0)
Alcohol consumption, *n* (%)		
Abstainer	94 (77.0)	93 (75.0)
≤28 units alcohol per week	28 (23.0)	31 (25.0)
>28 units alcohol per week	0	0
Height (cm), mean (SD)	166.6 (8.7)	165.4 (7.8)
Median (range)	167.0 (141, 183)	165.0 (143, 182)
Weight (kg), mean (SD)	72.51 (12.48)	71.87 (11.38)
Median (range)	71.75 (46.0, 124.5)	71.30 (50.0, 110.0)
Body mass index (kg/m^2^), mean (SD)	26.00 (3.15)	26.19 (3.22)
HbA1c (%), mean (SD)	7.90 (0.68)	7.87 (0.72)
Duration of diabetes (years)^a^, mean (SD)	5.05 (3.90)	5.41 (4.22)
Total daily metformin dose (mg), mean (SD)	1368.9 (341.4)	1392.9 (353.1)

Note

Percentages are based on the number of patients in each treatment group.

Abbreviations: HbA1c, glycosylated haemoglobin; SD, standard deviation.

^a^ Duration of diabetes (years) = (year of screening visit − year of diagnosis) + (month of screening visit − month of diagnosis)/12.

### 3.3 Outcomes

#### 3.3.1 Primary efficacy end‐point

**FIGURE 2:**
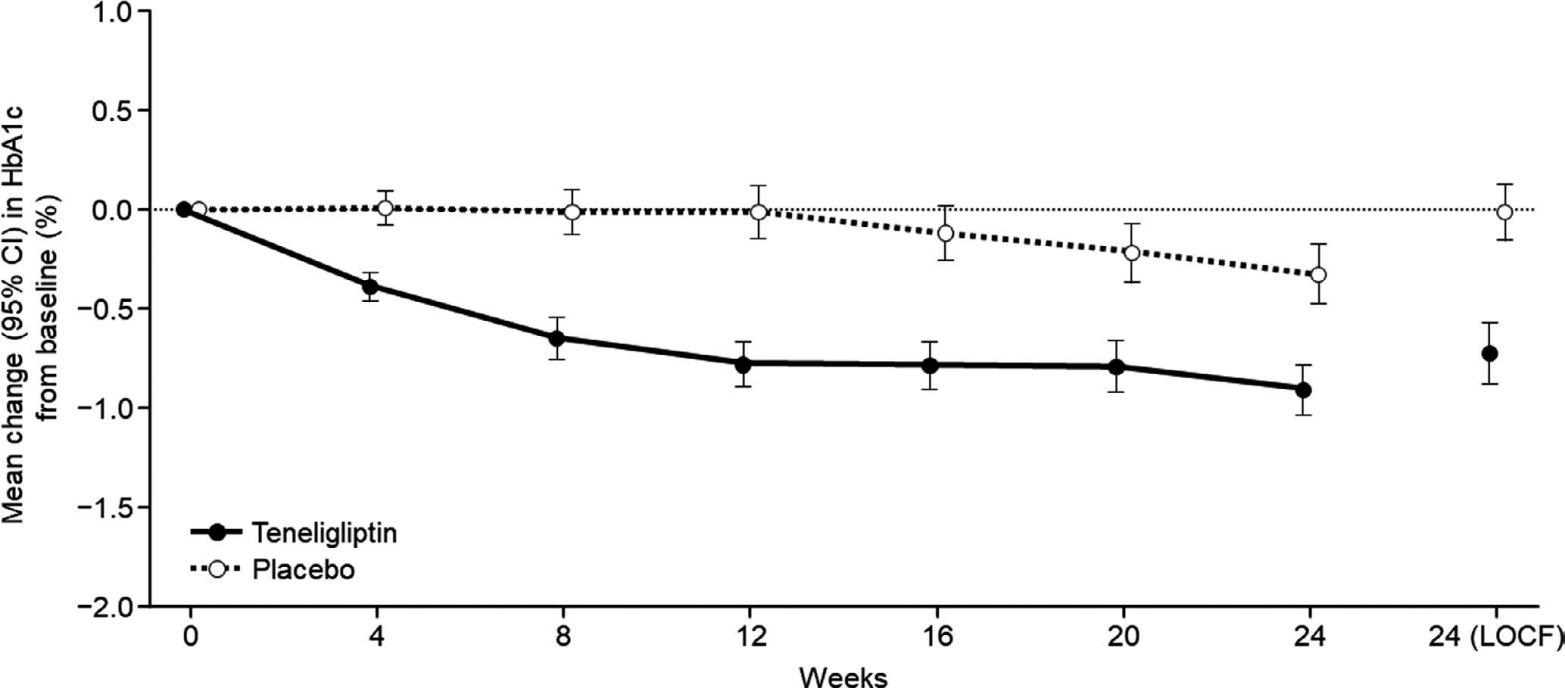
Mean change in HbA1c from baseline to Week 24 and Week 24 (LOCF) in the FAS. Baseline is defined as the most recent assessment prior to randomization. Missing HbA1c values at Week 24 were imputed using the LOCF method. CI, confidence interval; FAS, full analysis set; HbA1c, glycosylated haemoglobin; LOCF, last observation carried forward

The mean ± SD of HbA1c at Week 24 was 7.18 ± 1.02% and 7.85 ± 1.04% in the teneligliptin and placebo groups, respectively. Figure 2 shows the mean changes in HbA1c from baseline to Week 24 in both groups. The least square mean (LSM) change from baseline ± standard error [SE] was −0.72 ± 0.07% (95% CI: −0.87, −0.58) and −0.01 ± 0.07% (95% CI: −0.16, 0.13) in the teneligliptin and placebo groups, respectively. The difference (LSM ± SE) between groups was −0.71 ± 0.11% (95% CI: −0.92, −0.50), which was statistically significant in favour of teneligliptin (ANCOVA, *p* < .0001). A statistically significant result was also obtained in the sensitivity analysis (MMRM, *p* < .0001).

#### 3.3.2 Secondary efficacy end‐point

**FIGURE 3:**
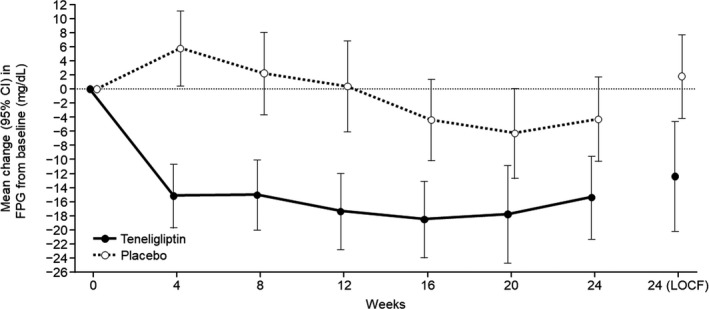
Mean change in fasting plasma glucose (FPG) from baseline to Week 24 and Week 24 (LOCF) in the FAS. Baseline is defined as the most recent assessment prior to randomization. Missing fasting plasma glucose values at Week 24 were imputed using the LOCF method. CI, confidence interval; FAS, full analysis set; FPG, fasting plasma glucose; LOCF, last observation carried forward

The mean ± SD FPG at baseline in the teneligliptin and placebo groups was 164.5 ± 35.8 and 170.8 ± 35.3 and at Week 24 was 152.1 ± 42.9 and 172.7 ± 43.2 mg/dl, respectively. Figure 3 shows the mean changes in FPG from baseline to Week 24 in both groups. The difference (LSM ± SE) between groups was −16.5 ± 4.7 mg/dl (95% CI: −25.7, −7.2), and this difference was statistically significant in favour of teneligliptin (ANCOVA, *p* = .0005).

#### 3.3.3 Other efficacy end‐points

The proportion of patients who achieved HbA1c <7.0% at Week 24 was 41.7% (48 of 115 patients) in the teneligliptin group and 16.1% (19 of 118 patients) in the placebo group. According to logistic regression analyses with treatment as a fixed effect and baseline HbA1c value as a covariate, the odds ratio of HbA1c <7.0% at Week 24 for teneligliptin was 5.33 (95% CI: 2.44, 11.65; *p* < .0001).

A statistically significant difference in favour of teneligliptin was also observed in change in HbA1c at Week 12 (LSM difference of −0.81 ± 0.09% [95% CI: −0.98, −0.64]; *p* < .0001). The difference (LSM ± SE) of the change in FPG from baseline to Week 12 between the placebo group and the teneligliptin group was −22.2 ± 3.9 mg/dl, and the difference between groups was significant in favour of teneligliptin (ANCOVA, *p* < .0001).

No significant differences were observed in the change in fasting insulin (*p* = .3709), fasting C‐peptide level (*p* = .2232), fasting glucagon level (*p* = .2197), HOMA‐IR (*p* = .8182) and HOMA‐β (*p* = .3605) from baseline to Week 24 between the placebo and the teneligliptin groups. The ANCOVA with treatment as a fixed effect and baseline body weight as a covariate showed that the difference (LS mean ± SE) of the change in body weight from baseline to Week 24 between the placebo group and the teneligliptin group was 0.33 ± 0.28 kg (95% CI: −0.22, 0.88), but the difference between groups was not significant (ANCOVA, *p* = .2364; Table S1).

#### 3.3.4 Subgroup analysis

Based on the subgroup analysis of change in HbA1c from baseline to Week 24 by baseline characteristics, including sex, age (50–60 and 60–70 years), metformin total daily dose 1000–<1500 mg and ≥1500 mg, alcohol consumption (abstainer and ≤28 units of alcohol per week), BMI 20–25 and 25−30 kg/m^2^, baseline HbA1c from 7% to 8% and from 8% to 9%, baseline FPG, and duration of diabetes, teneligliptin significantly reduced HbA1c compared with placebo in most of the subgroup analyses (Table S2). No significant differences between groups were observed for the age subgroups <40 years, 40–<50 years, and ≥70 years; BMI ≥30 kg/m^2^; and patients with baseline HbA1c <7.0% or ≥9.0%.

### 3.4 Safety

The safety analysis set consisted of 247 patients (124 patients in the teneligliptin group and 123 patients in the placebo group). TEAEs were observed in 73 patients (58.9%) in the teneligliptin group and 84 patients (68.3%) in the placebo group. The most frequent AEs by preferred term with an incidence ≥2% in any group were upper respiratory tract infection (11.3% and 17.9% in the teneligliptin and placebo groups, respectively), hyperuricaemia (8.9% and 10.6%, respectively) and hyperlipidaemia (6.5% and 5.7%, respectively; Table 2).

**TABLE 2:** Summary of adverse events, treatment‐emergent adverse events, and adverse drug reactions (safety analysis set) and treatment‐emergent adverse events by system organ class and preferred term with an incidence of ≥2%

	Teneligliptin (*N* = 124) *n* (%)	Placebo (*N* = 123) *n* (%)
TEAE	73 (58.9)	84 (68.3)
Severe TEAE^a^	1 (0.8)	4 (3.3)
Serious TEAE	4 (3.2)	6 (4.9)
ADR^b^	22 (17.7)	26 (21.1)
Serious ADR	3 (2.4)	3 (2.4)
Treatment‐emergent cardiovascular events	1 (0.8)	1 (0.8)
Study drug‐related treatment‐emergent cardiovascular events	1 (0.8)	1 (0.8)
TEAE leading to discontinuation	2 (1.6)	2 (1.6)
ADR leading to discontinuation	2 (1.6)	2 (1.6)
Hypoglycaemia	4 (3.2)	3 (2.4)
Study drug‐related hypoglycaemia	2 (1.6)	0
System organ class		
Preferred term^c^		
Infections and infestations	32 (25.8)	37 (30.1)
Upper respiratory tract infection	14 (11.3)	22 (17.9)
Bronchitis	4 (3.2)	1 (0.8)
Pharyngitis	3 (2.4)	1 (0.8)
Nasopharyngitis	2 (1.6)	4 (3.3)
Gingivitis	1 (0.8)	3 (2.4)
Metabolism and nutrition disorders	27 (21.8)	33 (26.8)
Hyperuricaemia	11 (8.9)	13 (10.6)
Hyperlipidaemia	8 (6.5)	7 (5.7)
Hypoglycaemia	4 (3.2)	3 (2.4)
Dyslipidaemia	4 (3.2)	2 (1.6)
Hyperkalaemia	1 (0.8)	3 (2.4)
Investigations	13 (10.5)	12 (9.8)
Protein urine present	5 (4.0)	2 (1.6)
Renal and urinary disorders	13 (10.5)	14 (11.4)
Proteinuria	7 (5.6)	5 (4.1)
Diabetic nephropathy	1 (0.8)	5 (4.1)
Gastrointestinal disorders	11 (8.9)	17 (13.8)
Diarrhoea	3 (2.4)	5 (4.1)
Chronic gastritis	0	4 (3.3)
Hepatobiliary disorders	9 (7.3)	8 (6.5)
Hepatic function abnormal	5 (4.0)	5 (4.1)
Musculoskeletal and connective tissue disorders	5 (4.0)	6 (4.9)
Blood and lymphatic system disorders	4 (3.2)	9 (7.3)
Leucopenia	3 (2.4)	4 (3.3)
Thrombocytopaenia	1 (0.8)	4 (3.3)
Cardiac disorder	4 (3.2)	5 (4.1)
Injury, poisoning and procedural complications	4 (3.2)	2 (1.6)
Nervous system disorders	4 (3.2)	7 (5.7)
Dizziness	2 (1.6)	3 (2.4)
Vascular disorders	3 (2.4)	10 (8.1)
Hypertension	3 (2.4)	8 (6.5)
Eye disorders	3 (2.4)	4 (3.3)
Endocrine disorders	3 (2.4)	1 (0.8)
Hyperglucagonaemia	3 (2.4)	1 (0.8)
Skin and subcutaneous tissue disorders	2 (1.6)	5 (4.1)

Notes

Percentages are based on the number of patients in each treatment group. Each patient is counted only once within each system organ class and within each preferred term.

Abbreviations: ADR, adverse drug reaction; AE, adverse event; *n* (%), number and percentage of patients affected; TEAE, treatment‐emergent adverse event.

^a^ The severity of an AE was graded by Investigator as 1 = mild, 2 = moderate and 3 = severe. If any AE occurred more than once, the highest severity was summarized. For AEs with missing severity, the most severe assessment was imputed.

^b^ ADRs were defined as AEs where the causal relationship to study drug was classified as a reasonable possibility. Any missing relationship of an AE to study drug was considered a reasonable possibility.

^c^ All AEs as described by the investigators (verbatim) were coded using MedDRA version 21.0.

Four patients (3.2%) in the teneligliptin and three patients (2.4%) in the placebo groups reported hypoglycaemia. Of these, two patients (1.6%) in the teneligliptin group presented study drug‐related hypoglycaemia. No severe hypoglycaemia events occurred during the study.

One patient (0.8%) each in the teneligliptin and placebo groups presented a cardiovascular event. The patient in the teneligliptin group had a cerebral infarction of mild severity, reported as not resolved/not recovered and possibly related to the study drug. The patient in the placebo group had a severe cerebral infarction, reported as resolved/recovered with sequelae and possibly related to the study drug.

Two patients (1.6%) in each of the teneligliptin and placebo groups reported at least one ADR leading to drug discontinuation: one event of arthralgia and one event of cerebral infarction in the teneligliptin group and one event of cerebral infarction and one event of diarrhoea in the placebo group. No AEs or ADRs resulted in deaths during the study. There were no notable changes or any differences between groups in laboratory values, vital signs, physical examination, or 12‐lead ECG parameters from baseline to Week 24.

## 4 DISCUSSION

Diabetes is estimated to affect >100 million Chinese individuals,^1^ and while metformin is the recommended first‐line pharmacologic therapy,^3^ monotherapy may become less effective at maintaining disease control over time.^4^ The combination of the DPP‐4 inhibitor teneligliptin plus metformin has been reported previously to be tolerable and to improve glucose control.^22^, ^23^ However, data relating to the use of this combination in Chinese patients are lacking. This is the first clinical trial to compare the efficacy and safety of teneligliptin versus placebo for type 2 diabetes patients inadequately controlled with metformin and lifestyle changes in China.

The present results demonstrate that teneligliptin once daily at a dose of 20 mg for 24 weeks in patients receiving stable metformin doses of ≥1000 mg/day led to significant reductions in HbA1c (−0.71%) and FPG (−16.5 mg/dl) levels compared with placebo, without any major safety concerns. These findings indicate that the 24‐week once‐daily administration of the addition of teneligliptin 20 mg to ongoing metformin therapy was effective in improving glucose control for Chinese patients.

At Week 24, a significantly greater proportion of patients achieved HbA1c <7.0% with teneligliptin (41.7%) compared with placebo (16.1%; *p* < .0001) while concomitantly receiving metformin. Additionally, teneligliptin led to significant changes in HbA1c (−0.81%; *p* < .0001) and FPG (−22.2 mg/dl; *p* < .0001) at Week 12. The present primary efficacy results resemble those reported in a previous European study.^22^ Teneligliptin administered concomitantly with metformin showed statistically significant reductions in HbA1c after 24 weeks (42.4% of patients achieved HbA1c <7.0% with teneligliptin compared with placebo [19.8%, *p* < .001] and change from baseline to Week 24 was −0.76% [*p* <.001]).^22^ Furthermore, the results of other efficacy measures (changes in HbA1c and FPG at Week 12) resemble the results of a phase 3 Asian trial evaluating teneligliptin combined with metformin in Korean patients with type 2 diabetes inadequately controlled with metformin. At 16 weeks, teneligliptin administration resulted in significant reductions in HbA1c (−0.78%) and FPG (−22.42 mg/dl) levels compared with placebo, which resembles our results at 24 weeks.^23^ The present efficacy findings resemble studies of other DPP‐4 inhibitors in combination with metformin.^19^, ^20^, ^21^, ^24^, ^25^ Specifically, when comparing the efficacy results of adding teneligliptin to ongoing metformin observed in this study with that of a similar study of another class 3 DPP‐4 inhibitor, sitagliptin, conducted in the United States in patients who were inadequately controlled with metformin monotherapy,^25^ the mean changes in HbA1c from baseline were similar for sitagliptin (−0.73%) and teneligliptin (−0.72%). Notably, the baseline HbA1c level in that study was 7.7%, which was slightly lower than that in our study (7.9%). Another similar trial conducted in Europe^20^ showed a reduction of −0.65% in HbA1c from baseline with sitagliptin added on to ongoing metformin therapy. Similar findings were reported with gemigliptin, another recently developed DPP‐4 inhibitor, with reductions in HbA1c of −0.77%.^24^ In these studies of other DDP‐4 inhibitors, as in this teneligliptin study, there was no increased risk of hypoglycaemia, gastrointestinal AEs or other AEs. Of note, several systematic reviews and meta‐analyses of DPP‐4 inhibitors have suggested that this class of drugs exhibits a greater glucose‐lowering efficacy in Asian patients compared with other ethnic groups, although the underlying mechanisms remain unclear.^26^, ^27^, ^28^ To date, the combination of teneligliptin and metformin has not been sufficiently studied in patients of different races or ethnicities to make similar claims,for example, no data in Japanese patients have yet been made available. Thus, further studies are warranted to examine whether the efficacy of treatment with teneligliptin and metformin may be affected by intrinsic patient demographic factors.

For some of the other efficacy measures, such as change in fasting insulin, fasting C‐peptide level, fasting glucagon level, and HOMA‐IR and HOMA‐β from baseline to Week 24, there were no significant differences between the placebo and the teneligliptin groups. Given the mechanism of action of teneligliptin,^29^ the effects on these measures may have been expected, however, the reason for the lack of observed differences between treatment groups is unknown.

There were no significant differences in body weight between the placebo and the teneligliptin groups. Of note, this lack of significant difference in body weight between the placebo and the teneligliptin groups signals to the possibility that teneligliptin is weight neutral, as reported previously.^30^

The incidence of hypoglycaemia was similar (3.2% and 2.4%) in both the teneligliptin and placebo groups. Among the four patients who reported hypoglycaemia in the teneligliptin group, hypoglycaemia (1.6%) was considered related to the study drug in only two patients. Moreover, the prevalence of hypoglycaemia in the teneligliptin group of the present study (3.2%) was similar to that reported in a Korean study (2.9%).^23^ The overall incidence of cardiovascular events was also similar in both groups (0.8%). There was no clear evidence of a relationship between cardiovascular events noted in the present study and teneligliptin. Over the years, concerns relating to pancreatic safety have been raised in regard to the use of DPP‐4 inhibitors,^31^ however, recent meta‐analyses have indicated that there is no relationship between use of DPP‐4 inhibitors and the development of pancreatic cancer, and that DPP‐4 inhibitors are associated with a low or negligible risk of acute pancreatitis.^32^, ^33^ No pancreatic TEAEs were observed in our study. Notably, very few patients discontinued due to AEs during the study, with the number of discontinuations being the same in both groups (two patients [1.6%] each). Thus, overall, teneligliptin 20 mg once daily added to ongoing metformin therapy was well tolerated during 24 weeks of treatment in the studied population.

In an additional subgroup analysis of change in HbA1c from baseline to Week 24 by baseline characteristics, teneligliptin significantly reduced HbA1c compared with placebo by background characteristics, suggesting possible improvements in HbA1c by teneligliptin regardless of patient baseline characteristics. However, no significant differences between groups were observed for patients <40, 40–<50 and ≥70 years; with a BMI ≥30 kg/m^2^; or with baseline HbA1c <7.0% and ≥9.0%. Altogether, the results of the present study indicate that teneligliptin is a suitable treatment option for Chinese type 2 diabetes patients whose blood glucose levels are not adequately controlled by metformin treatment in addition to diet and exercise, as well as for patients who have difficulty in taking other oral antihyperglycaemic drugs due to AEs.

### 4.1 Limitations

The main limitations of this study were the lack of an active comparator, the short treatment period (24 weeks) and limited generalizability to populations of other ethnicities. Further, safety and efficacy outcomes of teneligliptin in combination with other drugs for diabetes, or for comorbidities (eg, hypertension) need to be clarified. Finally, statistically significant results of non‐primary efficacy end‐points should be considered only as signals of possible treatment effects because alpha levels were not adjusted for multiple testing.

## 5 CONCLUSIONS

Teneligliptin once daily at a dose of 20 mg for 24 weeks, concomitantly administered with metformin, significantly decreased HbA1c and FPG levels compared with placebo in Chinese type 2 diabetes patients inadequately controlled with metformin therapy. The combination was well tolerated, and no new safety concerns were raised.

## CONFLICT OF INTEREST

Linong Ji has received personal fees from Mitsubishi Tanabe Pharma Development (Beijing) Co., Ltd. Yi Wang is an employee of Mitsubishi Tanabe Pharma Development (Beijing) Co., Ltd. Gen Takayanagi is an employee of Mitsubishi Tanabe Pharma Development America, Inc. The remaining authors have no conflicts of interest to declare.

## AUTHOR CONTRIBUTIONS

Linong Ji contributed to the design and implementation of the research and to the writing of the manuscript. Ling Li, Jianhua Ma, Xuefeng Li, Dongmei Li, Bangzhu Meng, Weiping Lu, Jiao Sun and Yanmei Liu contributed to the implementation of the research and to the writing of the manuscript. Gen Takayanagi contributed to the analyses of the clinical data and to the writing of the manuscript. Yi Wang contributed to the design of the research and to the writing of the manuscript. All authors approved the final version of the manuscript.

## Supporting information

Appendix S1

Fig S1

Table S1‐S2

## Data Availability

The data that support the findings of this study are available from the corresponding author, upon reasonable request.
