# Supplementary material for: Efficacy and safety of teneligliptin added to metformin in Chinese patients with type 2 diabetes mellitus inadequately controlled with metformin: A phase 3, randomized, double‐blind, placebo‐controlled study
Source: Endocrinol Diabetes Metab. 2021 Jan 20;4(2):e00222. doi: 10.1002/edm2.222 (PMC8029565; doi:10.1002/edm2.222)
Supplement: Supplementary file 1 — Appendix S1 [file EDM2-4-e00222-s002.docx]

**Supplementary Text**

Documented symptomatic hypoglycaemia was defined as an event during which typical symptoms of hypoglycaemia were accompanied by a measured plasma glucose concentration of ≤70 mg/dL (3.9 mmol/L).

Other exclusion criteria were history of cardiovascular, renal, hepatic, or neurological diseases; history of serious diabetic complications; history of malignancy; history of drug abuse and/or alcoholism (more than 28 units of alcohol per week); women or men of childbearing potential who did not agree to use appropriate contraception methods; pregnant or lactating women, or those planning to become pregnant; <75% treatment compliance with the investigational product (placebo) during the placebo run-in period; and history of joint pain with DPP-4 inhibitors.

**Statistical Methods**

Sample size calculation

The sample size was calculated based on the primary efficacy variable, using a two-sided t-test at a 0.05 significance level to have 90% power to detect a treatment difference of 0.5% in mean change in HbA1c from baseline at Week 24 with a common standard deviation of 1.0%, and assuming a drop-out rate of 30%. Additionally, the number of cases was calculated considering the National Medical Products Administration regulations, which requires that at least 100 pairs of subjects should be enrolled in a phase 3 study conducted in China.

Analytical populations

The full analysis set included all patients in the randomized set who received at least one dose of study medication during the double-blind treatment period and had at least one post-baseline efficacy observation. The safety analysis set included all patients in the randomized set who had received at least one dose of study medication during the double-blind treatment period.
