# Supplementary figures and images for: Efficacy and safety of teneligliptin added to metformin in Chinese patients with type 2 diabetes mellitus inadequately controlled with metformin: A phase 3, randomized, double‐blind, placebo‐controlled study
Source: Endocrinol Diabetes Metab. 2021 Jan 20;4(2):e00222. doi: 10.1002/edm2.222 (PMC8029565; doi:10.1002/edm2.222)

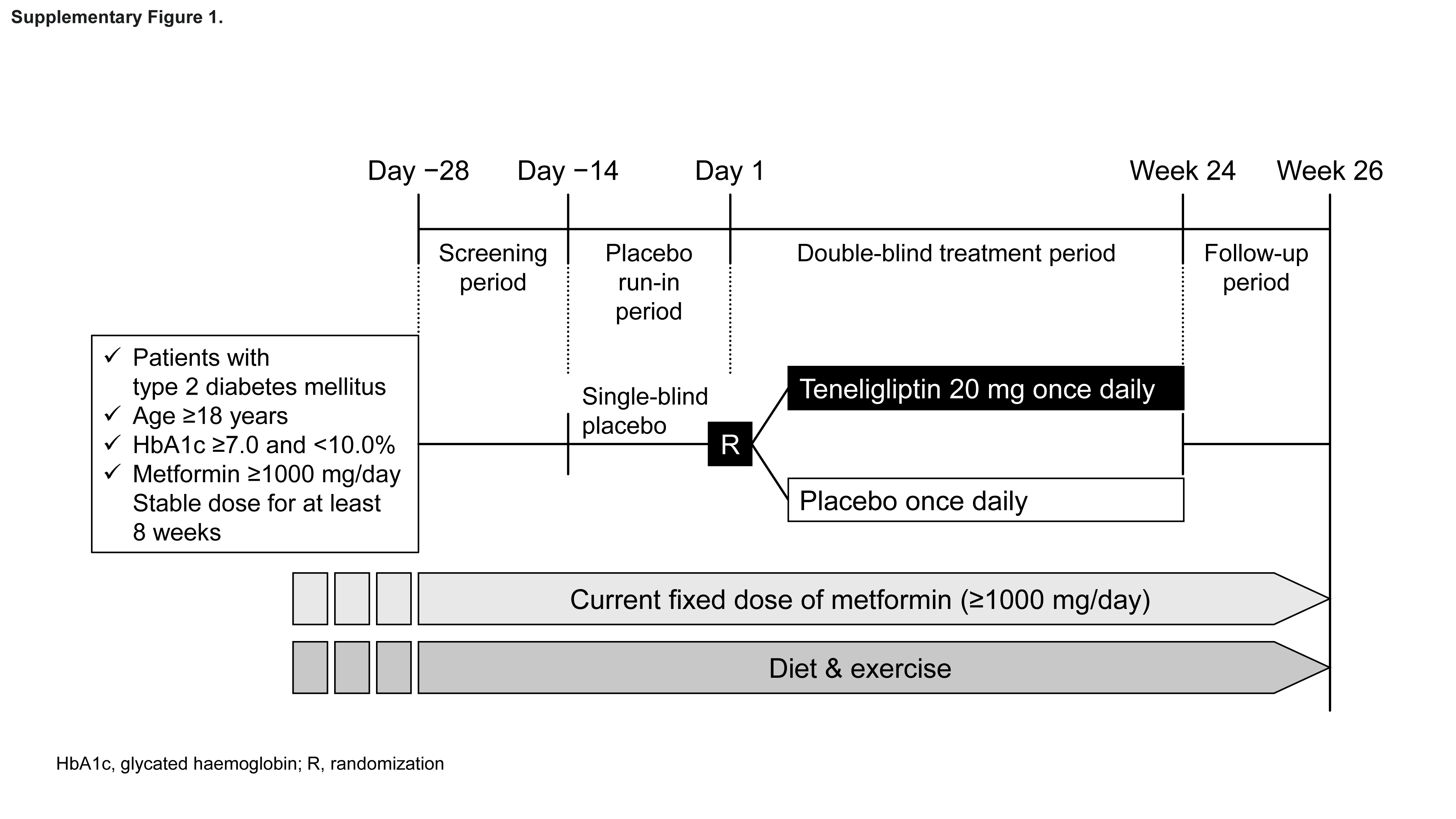

Supplement: Supplementary file 2 — Fig S1 [file EDM2-4-e00222-s001.tif]
