# Supplementary material for: Efficacy and safety of teneligliptin added to metformin in Chinese patients with type 2 diabetes mellitus inadequately controlled with metformin: A phase 3, randomized, double‐blind, placebo‐controlled study
Source: Endocrinol Diabetes Metab. 2021 Jan 20;4(2):e00222. doi: 10.1002/edm2.222 (PMC8029565; doi:10.1002/edm2.222)
Supplement: Supplementary file 3 — Table S1‐S2 [file EDM2-4-e00222-s003.docx]

**Supplementary Table 1. Summary of efficacy endpoints**

|  | **Baseline** | **Week 12** | **Week 24 (LOCF)** | **Change from baseline to Week 12** | **Change from baseline to Week 24 (ANCOVA using LOCF)** | | **Teneligliptin vs placebo at Week 24** | |
| --- | --- | --- | --- | --- | --- | --- | --- | --- |
|  | **Mean (SD)** | **Mean (SD)** | **Mean (SD)** | **Mean (SD)** | **LSM (SE)** | **95% CI** | **LSM difference (SE)** | **95% CI;**  **p value^†^** |
| **HbA1c (%)** |  |  |  |  |  |  |  |  |
| Placebo | 7.87 (0.72)  (N=124) | 7.94 (0.99) (N=124)^‡^ | 7.85 (1.04)  (N=124) | 0.08 (0.71) | −0.01 (0.07) | (−0.16, 0.13) | −0.71 (0.11) | (−0.92, −0.50)  <0.0001 |
| Teneligliptin | 7.90 (0.68)  (N=122) | 7.17 (0.87)  (N= 122)^‡^ | 7.18 (1.02)  (N=122) | −0.73 (0.64) | −0.72 (0.07) | (−0.87, -0.58) |  |  |
| **FPG (mg/dL)** |  |  |  |  |  |  |  |  |
| Placebo | 170.8 (35.3)  (N=124) | 172.9 (45.8)  (N=124)^‡^ | 172.7 (43.2)  (N=124) | 2.1 (35.8) | 3.0 (3.3) | (−3.5, 9.5) | −16.5 (4.7) | (−25.7, −7.2)  0.0005 |
| Teneligliptin | 164.5 (35.8)  (N=122) | 146.4 (30.0)  (N=122)^‡^ | 152.1 (42.9)  (N=122) | -18.1 (28.5) | −13.5 (3.3) | (−20.1, −7.0) |  |  |
|  |  |  |  |  |  |  |  |  |
| **Fasting insulin (μU/mL)** |  |  |  |  |  |  |  |  |
| Placebo | 8.475 (7.649)  (N=119) | 9.292 (12.022)  (N=101) | 8.489 (8.479)  (N=124) | 0.440 (5.452) | −0.228 (0.549) | (−1.309, 0.853) | 0.701 (0.781) | (−0.839, 2.240)  0.3709 |
| Teneligliptin | 9.356 (12.247)  (N=116) | 9.839 (10.626)  (N=108) | 9.649 (11.614)  (N=122) | 0.129 (6.610) | 0.473 (0.556) | (−0.622, 1.568) |  |  |
| **C-peptide (ng/mL)** |  |  |  |  |  |  |  |  |
| Placebo | 1.698 (0.687)  (N=124) | 1.666 (0.651)  (N=101) | 1.692 (0.745)  (N=124) | −0.059 (0.472) | −0.015 (0.050) | (−0.113, 0.083) | 0.086 (0.071) | (−0.053, 0.226)  0.2232 |
| Teneligliptin | 1.755 (0.728)  (N=122) | 1.882 (0.856)  (N=108) | 1.818 (0.740)  (N=122) | 0.114 (0.514) | 0.072 (0.050) | (−0.027, 0.171) |  |  |
| **Glucagon (pg/mL)** |  |  |  |  |  |  |  |  |
| Placebo | 3.93 (2.08)  (N=123) | 4.20 (2.46)  (N=101) | 4.10 (2.36)  (N=124) | 0.20 (1.80) | 0.14 (0.16) | (−0.17, 0.44) | 0.27 (0.22) | (−0.16, 0.71)  0.2197 |
| Teneligliptin | 4.45 (2.70)  (N=121) | 4.78 (3.15)  (N=108) | 4.84 (3.05)  (N=122) | 0.25 (1.83) | 0.41 (0.16) | (0.10, 0.72) |  |  |
| **HOMA-IR** |  |  |  |  |  |  |  |  |
| Placebo | 3.57 (3.13)  (N=119) | 3.96 (6.61)  (N=101) | 3.58 (3.49)  (N=124) | 0.43 (4.69) | −0.03 (0.19) | (−0.40, 0.34) | −0.06 (0.27) | (−0.59, 0.47)  0.8182 |
| Teneligliptin | 3.62 (3.69)  (N=116) | 3.43 (3.11)  (N=108) | 3.50 (3.36)  (N=122) | −0.28 (2.32) | −0.09 (0.19) | (−0.47, 0.29) |  |  |
| **HOMA-β** |  |  |  |  |  |  |  | (−11.99, 32.84)  0.3605 |
| Placebo | 32.12 (45.28)  (N=119) | 35.21 (39.48)  (N=101) | 35.73 (76.73)  (N=124) | −0.67 (32.74) | 3.10 (7.98) | (−12.62, 18.83) | 10.42 (11.38) |  |
| Teneligliptin | 43.48 (107.33)  (N=116) | 47.85 (92.96)  (N=108) | 56.08 (150.30)  (N=122) | 1.95 (77.43) | 13.53 (8.09) | (−2.40, 29.46) |  |  |
| **Body weight (kg)** |  |  |  |  |  |  |  |  |
| Placebo | 71.41 (11.44)  (N=124) | 71.37 (10.99)  (N=101) | 70.95 (11.51)  (N=120) | −0.38 (1.56) | −0.67 (0.20) | (−1.06, −0.28) | 0.33 (0.28) | (−0.22, 0.88)  0.2364 |
| Teneligliptin | 72.38 (12.47)  (N=122) | 72.29 (12.76)  (N=108) | 72.04 (12.30)  (N=119) | −0.12 (2.16) | −0.34 (0.20) | (−0.73, 0.05) |  |  |

Baseline is defined as the most recent assessment prior to randomization.

Missing values at Week 24 were imputed using LOCF method.

^†^Estimated based on ANCOVA method with continuous baseline value as covariate and treatment as fixed effect in the model.

‡Missing values at Week 12 were imputed using the LOCF method.

ANCOVA, analysis of covariance; CI, confidence interval; FPG, fasting plasma glucose; HbA1c, glycosylated hemoglobin; HOMA-β, homeostatic model assessment-beta; HOMA-IR, homeostatic model assessment-insulin resistance; LSM, least square mean; LOCF, last observation carried forward; SD, standard deviation; SE, standard error of the mean.

**Supplementary Table 2. Subgroup analyses of change in HbA1c from baseline to Week 24**

|  | **Baseline**  **Mean (SD)** | **Week 24 (LOCF)**  **Mean (SD)** | **Change from baseline to Week 24** | | **Difference** | | **p-value**^†^ |
| --- | --- | --- | --- | --- | --- | --- | --- |
|  |  |  | **LSM (SE)** | **95% CI** | **LSM (SE)** | **95% CI** |  |
| **Sex: male** |  |  |  |  |  |  |  |
| Placebo | 7.86 (0.79)  (N=67) | 7.81 (1.04)  (N=67) | −0.05 (0.10) | (−0.25, 0.16) | −0.63 (0.14) | (−0.91, −0.35) | <0.0001 |
| Teneligliptin | 7.82 (0.68)  (N=81) | 7.14 (1.05)  (N=81) | −0.68 (0.09) | (−0.86, −0.49) |  |  |  |
| **Sex: female** |  |  |  |  |  |  |  |
| Placebo | 7.87 (0.63)  (N=57) | 7.90 (1.04)  (N=57) | 0.03 (0.11) | (−0.18, 0.24) | −0.85 (0.17) | (−1.18, −0.52) | <0.0001 |
| Teneligliptin | 8.07 (0.66)  (N=41) | 7.24 (0.96)  (N=41) | −0.82 (0.13) | (−1.07, −0.57) |  |  |  |
| **Age group: <40 years** | |  |  |  |  |  |  |
| Placebo | 7.89 (0.64)  (N=11) | 7.95 (0.77)  (N=11) | 0.08 (0.43) | (−0.83, 1.00) | 0.33 (0.69) | (−1.15, 1.80) | 0.6456 |
| Teneligliptin | 7.73 (0.67)  (N=7) | 8.19 (2.09)  (N=7) | 0.41 (0.54) | (−0.74, 1.56) |  |  |  |
| **Age group: 40 to <50 years** | |  |  |  |  |  |  |
| Placebo | 7.80 (0.72)  (N=25) | 7.81 (1.08)  (N=25) | −0.01 (0.18) | (−0.37, 0.35) | −0.46 (0.27) | (−1.01, 0.08) | 0.0914 |
| Teneligliptin | 8.04 (0.78)  (N=20) | 7.54 (1.07)  (N=20) | −0.47 (0.20) | (−0.88, −0.07) |  |  |  |
| **Age group: 50 to <60 years** | |  |  |  |  |  |  |
| Placebo | 8.04 (0.77)  (N=46) | 7.94 (1.19)  (N=46) | −0.10 (0.11) | (−0.31, 0.12) | −0.82 (0.15) | (−1.12, −0.52) | <0.0001 |
| Teneligliptin | 7.81 (0.60)  (N=47) | 6.90 (0.73)  (N=47) | −0.91 (0.11) | (−1.12, −0.70) |  |  |  |
| **Age group: 60 to <70 years** | |  |  |  |  |  |  |
| Placebo | 7.61 (0.55)  (N=33) | 7.68 (0.86)  (N=33) | 0.06 (0.12) | (−0.17, 0.29) | −0.93 (0.16) | (−1.25, −0.62) | <0.0001 |
| Teneligliptin | 7.96 (0.71)  (N=41) | 7.08 (0.88)  (N=41) | −0.88 (0.10) | (−1.08, −0.67) |  |  |  |
| **Age group: ≥70 years** | |  |  |  |  |  |  |
| Placebo | 8.04 (0.90)  (N=9) | 8.03 (1.15)  (N=9) | 0.00 (0.29) | (−0.63, 0.63) | −0.38 (0.44) | (−1.33, 0.58) | 0.4086 |
| Teneligliptin | 7.94 (0.85)  (N=7) | 7.57 (1.07)  (N=7) | −0.38 (0.33) | (−1.10, 0.33) |  |  |  |
| **Alcohol consumption: abstainer** | |  |  |  |  |  |  |
| Placebo | 7.86 (0.69)  (N=93) | 7.91 (1.09)  (N=93) | 0.05 (0.09) | (−0.12, 0.23) | −0.69 (0.13) | (−0.94, −0.44) | <0.0001 |
| Teneligliptin | 7.88 (0.69)  (N=94) | 7.24 (1.10)  (N=94) | −0.64 (0.09) | (−0.82, −0.47) |  |  |  |
| **Alcohol consumption ≤ 28 units/week** | | |  |  |  |  |  |
| Placebo | 7.89 (0.81)  (N=31) | 7.68 (0.87)  (N=31) | −0.22 (0.11) | (−0.45, 0.01) | −0.77 (0.17) | (−1.11, −0.44) | <0.0001 |
| Teneligliptin | 7.97 (0.64)  (N=28) | 6.96 (0.66)  (N=28) | −0.99 (0.12) | (−1.23, −0.75) |  |  |  |
| **BMI: 20 to <25 kg/m^2^** | |  |  |  |  |  |  |
| Placebo | 7.80 (0.67)  (N=48) | 7.82 (1.08)  (N=48) | 0.02 (0.12) | (−0.21, 0.25) | −0.80 (0.17) | (−1.14, −0.46) | <0.0001 |
| Teneligliptin | 7.91 (0.74)  (N=41) | 7.13 (1.07)  (N=41) | −0.78 (0.13) | (−1.03, −0.53) |  |  |  |
| **BMI: 25 to <30 kg/m^2^** | |  |  |  |  |  |  |
| Placebo | 7.91 (0.75)  (N=57) | 7.93 (0.99)  (N=57) | 0.02 (0.10) | (−0.17, 0.21) | −0.81 (0.13) | (−1.06, −0.55) | <0.0001 |
| Teneligliptin | 7.89 (0.66)  (N=69) | 7.10 (0.79)  (N=69) | −0.79 (0.09) | (−0.96, −0.62) |  |  |  |
| **BMI: ≥30 kg/m^2^** | |  |  |  |  |  |  |
| Placebo | 8.02 (0.72)  (N=17) | 7.85 (1.11)  (N=17) | −0.18 (0.30) | (−0.80, 0.45) | 0.04 (0.48) | (−0.96, 1.04) | 0.9361 |
| Teneligliptin | 7.88 (0.55)  (N=11) | 7.74 (1.81)  (N=11) | −0.14 (0.38) | (−0.91, 0.64) |  |  |  |
| **Baseline HbA1c: <7.0%** | |  |  |  |  |  |  |
| Placebo | 6.73 (0.18)  (N=6) | 6.85 (0.23)  (N=6) | −0.05 (0.34) | (−0.81, 0.70) | 0.04 (0.49) | (−1.04, 1.13) | 0.9283 |
| Teneligliptin | 6.86 (0.08)  (N=7) | 6.70 (1.02)  (N=7) | −0.01 (0.31) | (−0.71, 0.69) |  |  |  |
| **Baseline HbA1c: 7.0 to <8.0%** | |  |  |  |  |  |  |
| Placebo | 7.45 (0.25)  (N=68) | 7.49 (0.79)  (N=68) | 0.03 (0.09) | (−0.16, 0.21) | −0.67 (0.13) | (−0.94, −0.41) | <0.0001 |
| Teneligliptin | 7.51 (0.29)  (N=67) | 6.85 (0.80)  (N=67) | −0.65 (0.09) | (−0.83, −0.46) |  |  |  |
| **Baseline HbA1c: 8.0 to <9.0%** | |  |  |  |  |  |  |
| Placebo | 8.33 (0.30)  (N=40) | 8.23 (0.98)  (N=40) | −0.07 (0.14) | (−0.35, 0.21) | −0.90 (0.20) | (−1.31, −0.50) | <0.0001 |
| Teneligliptin | 8.44 (0.28)  (N=39) | 7.51 (1.02)  (N=39) | −0.97 (0.14) | (−1.26, −0.69) |  |  |  |
| **Baseline HbA1c: ≥9.0%** | |  |  |  |  |  |  |
| Placebo | 9.49 (0.40)  (N=10) | 9.45 (0.94)  (N=10) | −0.08 (0.28) | (−0.68, 0.52) | −0.64 (0.42) | (−1.54, 0.26) | 0.1492 |
| Teneligliptin | 9.30 (0.19)  (N=9) | 8.53 (1.01)  (N=9) | −0.72 (0.30) | (−1.36, −0.08) |  |  |  |
| **Baseline FPG: <130 mg/dL** | |  |  |  |  |  |  |
| Placebo | 7.37 (0.76)  (N=6) | 7.37 (0.95)  (N=6) | −0.03 (0.22) | (−0.51, 0.45) | −0.71 (0.29) | (−1.33, −0.09) | 0.0280 |
| Teneligliptin | 7.51 (0.76)  (N=9) | 6.76 (0.57)  (N=9) | −0.74 (0.18) | (−1.13, −0.35) |  |  |  |
| **Baseline FPG: 130 to <160 mg/dL** | |  |  |  |  |  |  |
| Placebo | 7.50 (0.45)  (N=49) | 7.52 (0.77)  (N=49) | 0.00 (0.12) | (−0.24, 0.24) | −0.64 (0.16) | (−0.96, −0.31) | 0.0002 |
| Teneligliptin | 7.63 (0.56)  (N=57) | 6.98 (1.04)  (N=57) | −0.63 (0.11) | (−0.85, −0.41) |  |  |  |
| **Baseline FPG: 160 to <200 mg/dL** | |  |  |  |  |  |  |
| Placebo | 7.88 (0.63)  (N=43) | 7.68 (0.87)  (N=43) | −0.22 (0.11) | (−0.43, 0.00) | −0.68 (0.16) | (−1.00, −0.37) | <0.0001 |
| Teneligliptin | 7.99 (0.47)  (N=38) | 7.07 (0.68)  (N=38) | −0.90 (0.11) | (−1.13, −0.67) |  |  |  |
| **Baseline FPG: ≥200 mg/dL** | |  |  |  |  |  |  |
| Placebo | 8.64 (0.65)  (N=26) | 8.88 (1.16)  (N=26) | 0.23 (0.20) | (−0.17, 0.64) | −0.77 (0.31) | (−1.40, −0.13) | 0.0191 |
| Teneligliptin | 8.77 (0.58)  (N=18) | 8.23 (1.12)  (N=18) | −0.53 (0.24) | (−1.02, −0.05) |  |  |  |
| **Duration of diabetes: <1 year** | |  |  |  |  |  |  |
| Placebo | 7.60 (0.50)  (N=16) | 7.49 (0.96)  (N=16) | −0.11 (0.19) | (−0.50, 0.28) | −0.67 (0.26) | (−1.20, −0.15) | 0.0139 |
| Teneligliptin | 7.58 (0.74)  (N=19) | 6.80 (0.87)  (N=19) | −0.78 (0.17) | (−1.14, −0.43) |  |  |  |
| **Duration of diabetes: 1 to <5 years** | |  |  |  |  |  |  |
| Placebo | 7.88 (0.69)  (N=50) | 7.87 (1.06)  (N=50) | −0.01 (0.13) | (−0.26, 0.24) | −0.63 (0.18) | (−0.99, −0.27) | 0.0009 |
| Teneligliptin | 7.78 (0.61)  (N=47) | 7.15 (1.16)  (N=47) | −0.63 (0.13) | (−0.89, −0.37) |  |  |  |
| **Duration of diabetes: 5 to <10 years** | | |  |  |  |  |  |
| Placebo | 7.91 (0.77)  (N=38) | 8.04 (1.01)  (N=38) | 0.11 (0.13) | (−0.16, 0.38) | −0.88 (0.19) | (−1.25, −0.50) | <0.0001 |
| Teneligliptin | 8.17 (0.66)  (N=40) | 7.39 (1.01)  (N=40) | −0.77 (0.13) | (−1.03, −0.51) |  |  |  |
| **Duration of diabetes: ≥10 years** | |  |  |  |  |  |  |
| Placebo | 7.97 (0.83)  (N=20) | 7.75 (1.09)  (N=20) | −0.22 (0.17) | (−0.56, 0.12) | −0.56 (0.25) | (−1.07, −0.05) | 0.0332 |
| Teneligliptin | 7.96 (0.65)  (N=16) | 7.18 (0.60)  (N=16) | −0.78 (0.19) | (−1.16, −0.40) |  |  |  |
| **Metformin total daily dose: 1000 to** <**1500 mg** | |  |  |  |  |  |  |
| Placebo | 7.81 (0.71)  (N=46) | 7.85 (0.97)  (N=46) | 0.04 (0.10) | (−0.16, 0.24) | −0.90 (0.14) | (−1.17, −0.62) | <0.0001 |
| Teneligliptin | 7.83 (0.63)  (N=49) | 6.98 (0.81)  (N=49) | −0.86 (0.10) | (−1.05, −0.67) |  |  |  |
| **Metformin total daily dose:** ≥**1500 mg** | |  |  |  |  |  |  |
| Placebo | 7.90 (0.73)  (N=78) | 7.85 (1.09)  (N=78) | −0.05 (0.10) | (−0.25, 0.16) | −0.59 (0.15) | (−0.88, −0.29) | 0.0001 |
| Teneligliptin | 7.95 (0.71)  (N=73) | 7.31 (1.12)  (N=73) | −0.63 (0.11) | (−0.84, −0.42) |  |  |  |

^†^Estimated based on ANCOVA with baseline HbA1c value as co-variate and treatment as fixed effect in the model.

ANCOVA, analysis of covariance; CI, confidence interval; FPG, fasting plasma glucose; HbA1c, glycosylated hemoglobin; LSM, least square mean; LOCF, last observation carried forward; SD, standard deviation; SE, standard error of the mean.
